# Exploring Psychological Trends in Populations With Chronic Obstructive Pulmonary Disease During COVID-19 and Beyond: Large-Scale Longitudinal Twitter Mining Study

**DOI:** 10.2196/54543

**Published:** 2025-03-05

**Authors:** Chunyan Zhang, Ting Wang, Caixia Dong, Duwei Dai, Linyun Zhou, Zongfang Li, Songhua Xu

**Affiliations:** 1 The High School Affiliated to Xi'an Jiaotong University Xi'an China; 2 Institute of Medical Artificial Intelligence Second Affiliated Hospital of Xi'an Jiaotong University Xi'an China; 3 School of Electrical Engineering Xi'an Jiaotong University Xi'an China

**Keywords:** COVID-19, chronic obstructive pulmonary disease (COPD), psychological trends, Twitter, data mining, deep learning

## Abstract

**Background:**

Chronic obstructive pulmonary disease (COPD) ranks among the leading causes of global mortality, and COVID-19 has intensified its challenges. Beyond the evident physical effects, the long-term psychological effects of COVID-19 are not fully understood.

**Objective:**

This study aims to unveil the long-term psychological trends and patterns in populations with COPD throughout the COVID-19 pandemic and beyond via large-scale Twitter mining.

**Methods:**

A 2-stage deep learning framework was designed in this study. The first stage involved a data retrieval procedure to identify COPD and non-COPD users and to collect their daily tweets. In the second stage, a data mining procedure leveraged various deep learning algorithms to extract demographic characteristics, hashtags, topics, and sentiments from the collected tweets. Based on these data, multiple analytical methods, namely, odds ratio (OR), difference-in-difference, and emotion pattern methods, were used to examine the psychological effects.

**Results:**

A cohort of 15,347 COPD users was identified from the data that we collected in the Twitter database, comprising over 2.5 billion tweets, spanning from January 2020 to June 2023. The attentiveness toward COPD was significantly affected by gender, age, and occupation; it was lower in females (OR 0.91, 95% CI 0.87-0.94; *P*<.001) than in males, higher in adults aged 40 years and older (OR 7.23, 95% CI 6.95-7.52; *P*<.001) than in those younger than 40 years, and higher in individuals with lower socioeconomic status (OR 1.66, 95% CI 1.60-1.72; *P*<.001) than in those with higher socioeconomic status. Across the study duration, COPD users showed decreasing concerns for COVID-19 and increasing health-related concerns. After the middle phase of COVID-19 (July 2021), a distinct decrease in sentiments among COPD users contrasted sharply with the upward trend among non-COPD users. Notably, in the post-COVID era (June 2023), COPD users showed reduced levels of joy and trust and increased levels of fear compared to their levels of joy and trust in the middle phase of COVID-19. Moreover, males, older adults, and individuals with lower socioeconomic status showed heightened fear compared to their counterparts.

**Conclusions:**

Our data analysis results suggest that populations with COPD experienced heightened mental stress in the post-COVID era. This underscores the importance of developing tailored interventions and support systems that account for diverse population characteristics.

## Introduction

On January 30, 2020, the World Health Organization declared the COVID-19 pandemic as a public health emergency of international concern [1]. Three years later, on May 5, 2023, given the encouraging trends observed in the COVID-19 statistics, the World Health Organization determined that COVID-19 no longer constituted a public health emergency of international concern [2]. However, this did not indicate that COVID-19 was no longer a global health threat [3]. The COVID-19 pandemic has had and continues to exert a profound and long-lasting impact on human lives worldwide, affecting public health, food systems, and global job markets [4,5]. In this situation, populations with chronic diseases, particularly chronic obstructive pulmonary disease (COPD), face great hardships [6,7] due to several reasons. First, patients with COPD are more likely to develop severe COVID-19 symptoms due to their respiratory illness [8]. Second, the overwhelming demands of managing COVID-19 may strain valuable health care resources, potentially affecting populations with COPD due to resource allocation [6]. Third, COVID-19 has greatly disrupted the daily lives of patients with COPD, leading to a higher risk of exacerbations and even mortalities. However, in contrast to the tangible physical consequences, the long-term effects of COVID-19 on the psychological conditions of patients with COPD have not drawn sufficient attention.

There are limited studies on the psychological effects of COVID-19 on patients with COPD (Table S1 in Multimedia Appendix 1) [9-14]. These studies have highlighted the high rates of psychological stress, including depression, anxiety, and fear, among patients with COPD under the threat of COVID-19. However, all these studies focused primarily on the early acute phase of COVID-19, thereby providing limited insight into the mental conditions of patients with COPD over the long term. Drawing parallels from the historical context of the SARS pandemic, wherein distinct chronological phases were discerned in the psychological conditions of SARS survivors [15], the psychological aftermath of COVID-19 survivors may exhibit similar enduring and evolving traits over the long term. Furthermore, surveys in the previous studies were conducted through traditional means, including telephone [10-12,14], video calls [14], interviews [13], and questionnaires [10,12], requiring substantial labor resources and physical contacts. This ultimately limited the amount of data that could be collected during the pandemic.

In recent years, social media data mining has been widely used in health-related domains—both physical and mental. In the physical health domain, for example, Pradeepa et al [16] proposed a methodology to identify various symptoms associated with stroke and preventive measures for stroke from social media data. Fu et al [17] conducted a scoping review of methodologies for analyzing social media content in health care research and categorized health care areas into health practice, health services, and health education based on social media. In the mental health domain, for capturing the prevailing psychological conditions of the public during the COVID-19 pandemic, social media data mining has proven to be an effective solution. Compared with conventional survey approaches, social media data mining has significantly higher efficiency while minimizing infection risks by circumventing the need for physical contact. More importantly, the richness of social media data facilitates the discernment of long-term trends in emotional changes within a specific time period. For example, Lwin et al [18] used Twitter data to explore global trends in the 4 emotions—fear, anger, sadness, and joy—alongside their relative salience, spanning from January 28 to April 9, 2020. Similarly, through mining large-scale Twitter data, Zhang et al [19] conducted a cross-sectional study on web-based public sentiments in the early phase of the COVID-19 pandemic. However, these studies tended to either treat the populace as a monolithic entity, thus disregarding possible disparities among various population groups or only focus on general users while ignoring the existence of certain specific populations.

Considering the limitations of existing studies [9-19], this study aims to investigate the long-term psychological trends and patterns of populations with COPD with different characteristics during and after the COVID-19 pandemic. A new deep learning framework has been proposed to intelligently extract and analyze the characteristics of populations with COPD based on large-scale Twitter data.

## Methods

### Study Design

**Figure 1:**
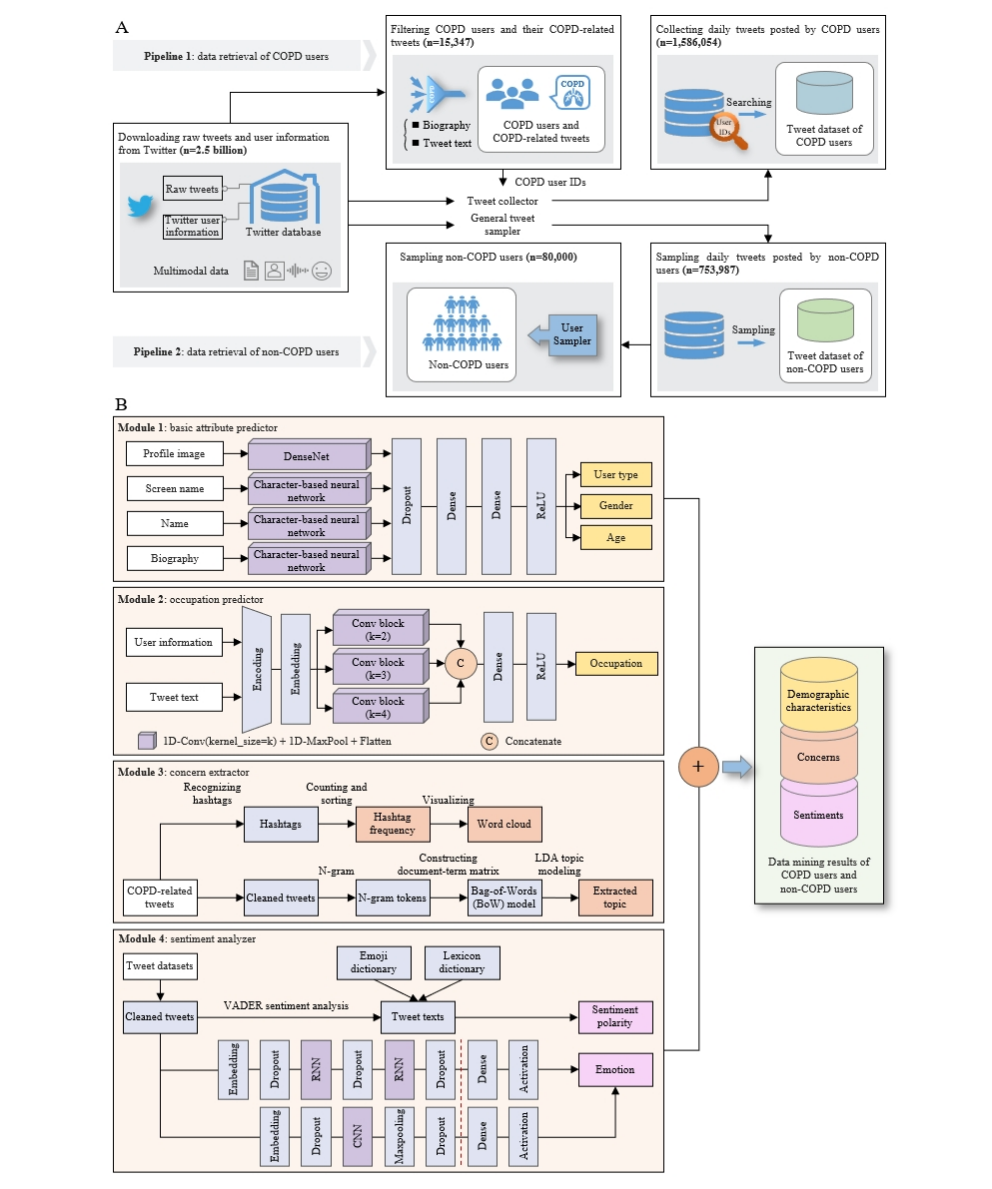
Overall design of the Deep-COPD framework. (A) Data retrieval procedure. (B) Data mining procedure. CNN: convolutional neural network; Conv: convolutional; COPD: chronic obstructive pulmonary disease; LDA: latent Dirichlet allocation; RNN: recurrent neural network; ReLU: rectified linear unit; VADER: valence aware dictionary and emotional reasoner.

This study was conducted following the STROBE (Strengthening the Reporting of Observational Studies in Epidemiology) guidelines [20] (Multimedia Appendix 2). In this study, raw Twitter data were collected with our self-designed program via the Twitter application programming interface [21]. These data are composed of multimodal information, including texts, emojis, images, and hashtags and are stored in a chronological order in our Twitter database. To date, over 2.5 billion tweets have been collected, spanning from January 2020 to June 2023. Based on these data, we designed a 2-stage framework, titled Deep-COPD, incorporating a series of deep learning algorithms to track COPD Twitter users (hereafter referred to as COPD users). The flowcharts for the data retrieval (stage 1) and data mining (stage 2) procedures are shown in Figure 1.

### Data Retrieval

Figure 1A shows the procedure of data retrieval, which involves 2 pipelines: pipeline 1, retrieving the data of COPD users, including user information, COPD-related tweets, and their daily tweets; and pipeline 2, retrieving the data of non-COPD users, including user information and their daily tweets. In pipeline 1, we used a filter pattern implemented as a regular expression composed of a set of COPD keywords to identify COPD users and tweets by searching 2 sources: user biographies and tweet texts (see Multimedia Appendix 1). The specific construction process of this regular expression can be found in the build_match_word_regex() function of the filter_copd.py file (lines 17-35) in the stage 1 folder of our GitHub repository. We collected the daily tweets posted by these COPD users through their user IDs. To limit the size of the data retrieved in pipeline 2, we sampled the tweets posted by non-COPD users at a rate of 0.05%. Through data retrieval, we obtained raw data that were used for the sentiment and psychological analysis. Moreover, we extracted data representing COPD and non-COPD characteristics from the daily tweets, enabling targeted research on the psychological evolution patterns associated with these 2 distinct groups. Specifically, this approach enables a more focused investigation into how the psychological trends may differ between users with COPD and those without COPD, thereby providing valuable insights into the impact of the condition on mental health over time. By analyzing these 2 groups separately, we aim to uncover specific psychological patterns and trends that could inform tailored interventions and support strategies for each population.

### Data Mining

The data mining procedure aims to extract distinct information from multimodal Twitter data by using a variety of deep learning algorithms, including natural language processing, image processing, and symbol processing (Figure 1B). It consists of the following 4 parallel intelligent modules. Module 1 and module 2 are used to extract demographic characteristics, including user type, gender, age, and occupation, from both COPD and non-COPD users. Module 3 extracts concern information from COPD-related tweets, while module 4 extracts sentiment information from the daily tweet datasets of both COPD and non-COPD users.

#### Module 1: Basic Attribute Predictor

Module 1 is an attribute predictor that predicts the probabilities of 3 demographic attributes, that is, user type, gender, and age, through the analysis of profile images, screen names, display names, and biographies. It was implemented with an open-source package of the M3 (multimodal, multilingual, and multiattribute) model [22], a multimodal deep neural system trained on a massive dataset. It is mainly composed of a dense convolutional network (DenseNet) [23] for image processing, 3 character-based neural networks for text processing, and 2 fully connected dense layers for result classification. The training and validating M3 model uses 5 distinct datasets: a large dataset of Twitter profiles whose gender and age are heuristically identified, a curated dataset of Twitter accounts belonging to organizations, an image dataset of faces from IMDb (internet movie database) and Wikipedia, a massive unlabeled set of users, and a crowdsourced dataset for all 3 attributes. For more detailed information, readers can refer to the original literature [22]. This module was tested with a subset of our tweet dataset, achieving the following benchmark performance: 99.07% accuracy for user type, 95.88% accuracy for gender, and 77.65% accuracy for age attributes.

#### Module 2: Occupation Predictor

Module 2 is a self-designed deep learning model that derives the 2-class occupations of Twitter users from their profile information and tweet texts. It employs a word-based convolutional neural network that consists of an encoding layer, an embedding layer, 3 parallel convolutional blocks with different kernel sizes, and a dense layer. The training and validation data are obtained from a publicly available dataset [24] with 5191 Twitter users annotated with 9 occupation categories (OCs), which are defined according to the standard occupation classification [25]. In our study, the 9 OCs were merged into 2 OCs, abbreviated as OC1 and OC2 (see Table S4 in Multimedia Appendix 1). Individuals in OC1 are those of relatively higher socioeconomic status (such as managers, directors, and senior officials), and individuals in OC2 are those of relatively lower socioeconomic status (such as process, plant, and machine operatives). Using a 10-fold cross-validation approach, this module achieved an accuracy of 93.15% on the 2-class occupational dataset.

#### Module 3: Concern Extractor

Module 3 is a concern extractor that captures hashtags and latent Dirichlet allocation (LDA) [26] topics. Hashtags were identified in the tweet texts using a regular expression (#+[a-zA-Z0-9\-_]{1,}), and then the frequencies of these hashtags were calculated. LDA topic modeling was employed to identify the main topics expressed in the tweets. The original tweets underwent preprocessing, n-gram processing, and document-term matrix constructing, yielding a bag-of-words, which was fed into LDA models. The coherence score calculated by Gensim CoherenceMode [27] was used to evaluate the quality of the extracted topics. To pinpoint the optimal number of LDA topics, we performed a grid search within a range of 2-20 with a step of 1 for each year. The selection principle is to choose the number that makes the coherence score of the extracted topics reach the highest value. Through searching, the optimal number of topics and the coherence score for each year were obtained, as shown in Table S5 in Multimedia Appendix 1.

#### Module 4: Sentiment Analyzer

Module 4 is composed of a sentiment analyzer and an emotion detector to perceive sentiments from 2 different perspectives. The sentiment analyzer utilizes a widely used open-source tool known as the valence aware dictionary and emotional reasoner (VADER) [28]. VADER requires no training data, but it is constructed from a generalizable, valence-based, human-curated gold standard sentiment lexicon. It is highly effective in analyzing social media sentiments, achieving an *F*_1_ classification accuracy of 96%, outperforming human raters of 84% at classifying the sentiment of tweets. The emotion detector was built upon an open-source algorithm for emotion recognition on Twitter, which employs a character-based trained recurrent neural network [29]. The training and validation dataset is a set of tweets containing emotional hashtags and satisfying filtering requirements that searched from 73 billion tweets. Specifically, this study adopted the well-known Plutchik’s emotion wheel [30] model, which consists of 8 basic emotions: joy, sadness, fear, anger, trust, disgust, surprise, and anticipation. This emotion detector achieved a macro *F*_1_-score of 0.6950 for Plutchik’s emotion model [29].

Specifically, the deep learning algorithms chosen in this study were selected for their strengths in handling various data types. DenseNet was employed for image processing, while character-based and word-based neural networks were used for the text classification of demographic attributes and occupations. LDA’s topic modeling capability made it suitable for extracting concerns from large text datasets. VADER’s high accuracy ensured robust social media sentiment detection. Additionally, a character-based recurrent neural network enabled the emotion detector to effectively capture and classify complex emotional states.

The data mining procedure extracts valuable information from multimodal Twitter data by using advanced deep learning algorithms. This process involves 4 parallel intelligent modules, of which the outputs are as follows: module 1 (basic attribute predictor) predicts demographic attributes such as user type, gender, and age by analyzing user profile images, names, and biographies; module 2 (occupation predictor) classifies Twitter users’ occupations into 2 groups based on their profiles and tweet content; module 3 (concern extractor) identifies user concerns by extracting hashtags and topics by using LDA; and module 4 (sentiment analyzer) analyzes sentiments from tweets by using VADER for general sentiments and a character-based recurrent neural network for emotion detection. Together, these modules provide a comprehensive analysis of the demographic, psychological, and emotional factors affecting both COPD and non-COPD Twitter users.

The outputs from data retrieval and data mining are crucial for assessing the psychological trends in populations with COPD, particularly the changes in sentiments, prevalent emotions, and evolving concerns (eg, health-related topics) during and after COVID-19. Through data retrieval, raw Twitter data are collected, allowing for data extraction of COPD and non-COPD groups, which enables focused analysis of psychological patterns within the target population. The data mining process further analyzes these data, extracting key demographic attributes, concerns, and sentiments by using deep learning algorithms. The concern extraction identifies health-related topics, while the sentiment and emotion analyzer tracks shifts in emotional states and sentiments, providing critical insights into the psychological evolution of populations with COPD in response to the pandemic. This comprehensive data processing directly supports the study’s goal of understanding the psychological trends of COVID-19 in populations with COPD.

### Data Analysis Tools

Based on the data mining results from our Deep-COPD framework, we conducted attentiveness analysis, concern analysis, and sentiment analysis. In these analyses, multiple statistical tools were used, namely, odds ratio (OR), difference-in-difference (DiD), and emotion pattern method.

#### Statistical Measure OR

OR is a statistical measure widely used in medical literature to evaluate the relevance between an event (such as lung cancer) and a particular exposure (such as smoking) [31]. In this study, the event refers to COPD, and the exposure factors include gender, age, and occupation. The ORs are used to quantitatively assess the levels of attentiveness toward COPD under these different exposure factors (Table 1).

**Table 1:** A 2x2 table for the chronic obstructive pulmonary disease event and the gender exposure factor (a, b, c, and d represent the number of users).

	Chronic obstructive pulmonary disease event	No event of chronic obstructive pulmonary disease
Female exposure	a	b
Male exposure	c	d

Taking gender as the exposure factor as an example, the 2×2 table shown in Table 1 is used to calculate the attentiveness OR toward a COPD event in females compared to males. The formula is as follows: OR = (a/b)/(c/d) = ad/bc

An OR of 1 indicates that the gender factor does not affect the level of attentiveness toward COPD. When OR>1, it suggests that the attentiveness toward COPD is higher in females than in males. Conversely, when OR<1, it indicates that the attentiveness toward COPD is lower in females than in males. The confidence interval in OR provides a range of values within which the true OR is likely to fall, offering an indication of the precision and reliability of the estimate. As a typical value, 95% CI is commonly used, indicating that there is a 95% probability that the true OR lies within the specified range [31]. The following formula is used for 95% CI:



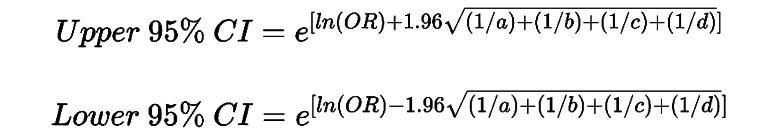



In addition, the *P* values were obtained using the chi-square test to evaluate the significance levels of the differences between groups under different exposure factors. A lower *P* value indicates a more statistically significant difference between the groups [32].

#### DiD Model

The DiD model is typically used to estimate the effect of a specific intervention or treatment by comparing the changes in outcomes over time between a population that is enrolled in a program (the treated group) and a population that is not (the control group) [33,34]. In this study, we took COPD users as the treated group and non-COPD users as the control group and adopted the DiD model to estimate the relative sentiment trends of COPD users. The formula of the DiD model is as follows:



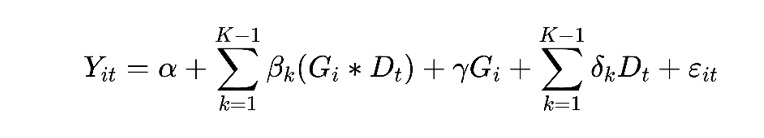



where *Y_it_* is the dependent variable representing the sentiment status for group *G_i_* and time *t*; *G_i_* is a dummy variable for the treated group (*G_0_*) or control group (*G_1_*); *D_t_* is a dummy variable for time *t*; *α, β_k_*, *γ*, *δ_k_* are the regression parameters; *K* is the total time period (*k*∈[1, *K*]); and *ε_it_* is an error item.

The coefficients of interest are those of the interaction term, *β_k_*, which can reflect the relative sentiment trends of COPD users compared to those of non-COPD users. A value of *β_k_* = 0 signifies that both groups have parallel sentiment trends, whereas a negative value indicates the extent to which the sentiment trend of the COPD group declines compared to that of the non-COPD group and vice versa.

#### Emotion Pattern

We proposed an emotion pattern method to calculate the values of the sentiment components of Plutchik’s 8 emotions. The emotion pattern of population p is composed of the sentiment component scores of Plutchik’s 8 emotions, which is expressed as follows:



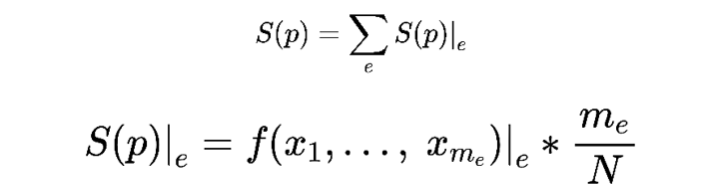



where *e*∈[joy, trust, surprise, anticipation, sadness, disgust, anger, fear], S (p)│_e_ is the sentiment component of population *p* on emotion *e*,



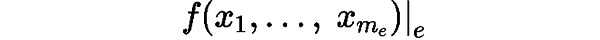



is the calculated sentiment scores of tweets



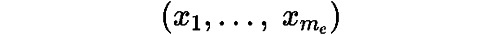



expressing emotion *e* via the VADER method *f*, *m_e_* is the number of tweets expressing emotion *e*, and *N* is the total number of tweets from population *p*.

### Ethical Considerations

This analysis did not receive approval from an institutional research board. Analysis of large bodies of text written in social media such as the Twitter social network is generally not considered human subjects research [35], and therefore, ethics approval was not sought. We confirm that all data used in this study have been deidentified, and no individual user is identifiable in any of the images included in the manuscript or supplementary data.

## Results

### Demographic Characteristics of the Participants

In total, 15,347 COPD users were identified as participants in this study. From this cohort, 1,586,054 daily tweets were retrieved, spanning from January 2020 to June 2023. For comparison, 80,000 non-COPD users and 753,987 general tweets were also selected in the same period. Table 2 presents the statistical data on the demographic characteristics of these two groups. To obtain a comprehensive understanding of the psychological conditions of populations with COPD during COVID-19 and beyond, we analyzed the data mined by the Deep-COPD framework along 3 dimensions: attentiveness analysis, concern analysis, and sentiment analysis.

**Table 2:** Statistical data of chronic obstructive pulmonary disease and non–chronic obstructive pulmonary disease users.

Characteristics			COPD^a^ users (n=15,347), n (%)		Non-COPD users (n=80,000), n (%)
**User type**					
	Individual	13,375 (87.15)		75,468 (94.34)	
	Organization	1972 (12.85)		4532 (5.67)	
**Gender**					
	Male	7854 (58.72)		42,509 (56.33)	
	Female	5521 (41.28)		32,959 (43.67)	
**Age (years)**					
	≤18	1179 (8.81)		29,810 (39.50)	
	19-29	2373 (17.74)		25,086 (33.24)	
	30-39	2091 (15.63)		8551 (11.33)	
	≥40	7732 (57.81)		12,021 (15.93)	
**Occupation category**					
	OC1^b^	7356 (55)		50,566 (67)	
	OC2^c^	6019 (45)		24,902 (33)	

^a^COPD: chronic obstructive pulmonary disease.

^b^OC1: occupation category 1 (relatively higher socioeconomic status such as managers, directors, and senior officials).

^c^OC2: occupation category 2 (relatively lower socioeconomic status such as process, plant, and machine operatives).

### Attentiveness Analysis

**Figure 2:**
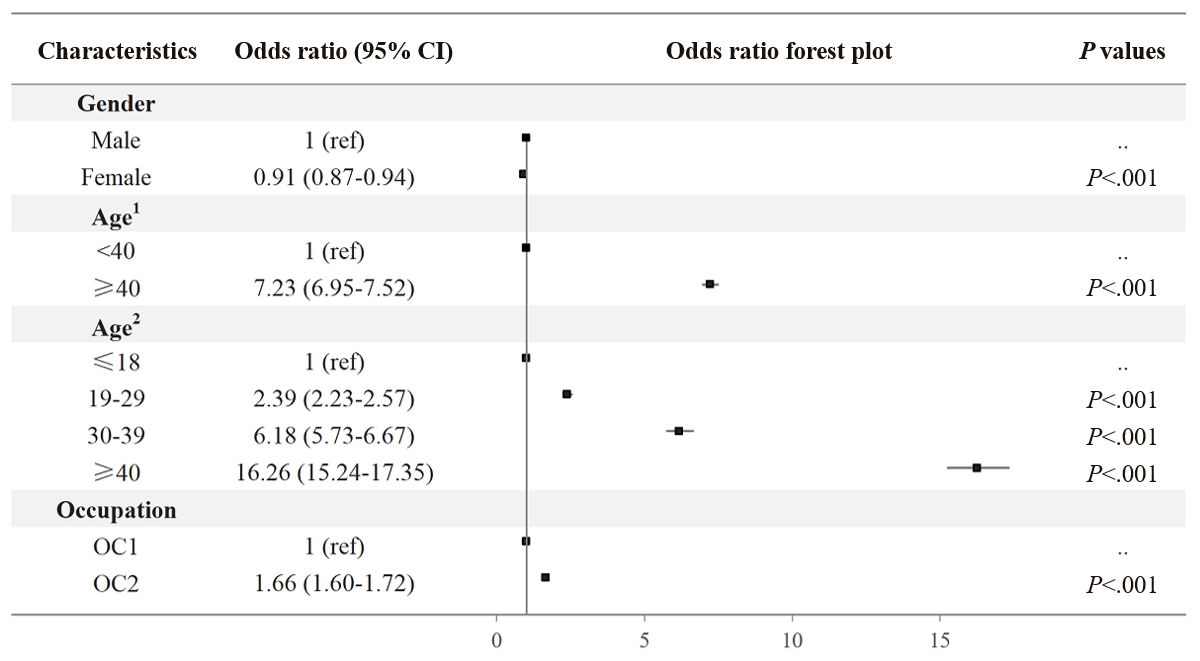
Attentiveness odds ratios toward chronic obstructive pulmonary disease in different demographic groups. Age 1: 2 age groups; age 2: 4 age groups; OC: occupation category; ref: reference.

Based on the statistics in different demographic groups (Table 2), a comparative study was performed between COPD and non-COPD users. As mentioned in the data analysis tools, we employed ORs to quantitatively assess the levels of attentiveness toward COPD in different groups (Figure 2). The ORs were calculated between COPD and non-COPD users under different exposure factors, including gender, age, and occupation. Results showed that the attentiveness toward COPD in females (OR 0.91, 95% CI 0.87-0.94; *P*<.001) was lower than that of males. The cohort aged 40 years and older exhibited a high OR of 7.23 (95% CI 6.95-7.52; *P*<.001), which was significantly higher than those younger than 40 years, and it showed a significant increase with age across 4 age groups. As for occupation, users in OC2 occupations exhibited a notably higher OR of 1.66 (95% CI 1.60-1.72; *P*<.001) compared to those in OC1 occupations. These results suggested that gender, age, and occupation are 3 important factors influencing attentiveness. Specifically, older male users with lower socioeconomic status (OC2) were the most concerning group.

### Concern Analysis

**Figure 3:**
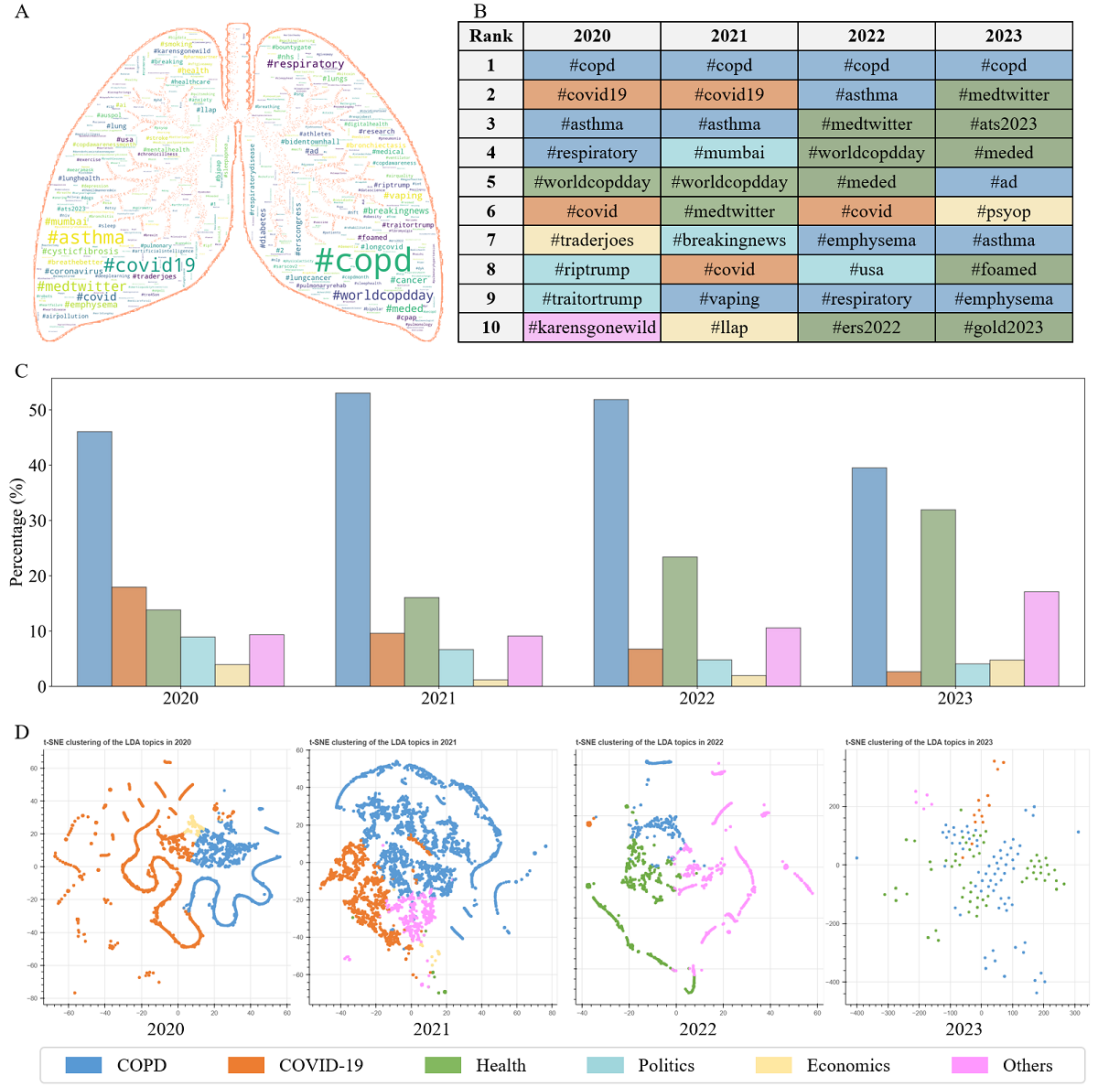
Results of the concern analysis. (A) Word cloud of the top 500 hashtags. (B) Top 10 most used hashtags in 4 years. (C) The percentages of the 6 categories of hashtags in 4 years. (D) Visualization of the latent Dirichlet allocation topics via t-distributed stochastic neighbor embedding clustering. COPD: chronic obstructive pulmonary disease; LDA: latent Dirichlet allocation; tSNE: t-distributed stochastic neighbor embedding clustering.

The word cloud of the top 500 hashtags in the tweets shows that #COPD and #COVID-19 were the most frequently used hashtags throughout the 4 years (Figure 3A). Among the top 10 hashtags in each year, #COPD consistently remained the primary concern throughout the 4-year period (Figure 3B). In 2020 and 2021, #COVID-19 ranked second but later gave way to health-related hashtags such as #medtwitter and #meded. To study the trends in the evolving concerns of COPD users, we categorized the 100 most frequently used hashtags from each year into 6 categories: COPD, COVID-19, health, politics, economics, and others. It is evident that the COPD category maintained the highest prevalence among COPD users, peaking at over 50% in 2021 (Figure 3C). The COVID-19 category held the second position in 2020, accounting for nearly 20%, but substantially decreased in 2021. On the other hand, the health category experienced continuous growth over the 4-year period, surpassing 30% in 2023 (Figure 3C). Figure 3D shows that the concerns from the perspective of LDA topics (see detailed topics in Table S6 in Multimedia Appendix 1) over the years follow the same trends as the hashtags. These statistical results highlight that, among COPD users, personal health became an even more significant concern as the pandemic receded.

### Sentiment Analysis

To gain an insight into the long-term trends and disparities of sentiments among different groups, we conducted a sentiment analysis spanning from January 2020 to June 2023. The sentiment score, calculated by VADER, was used to quantify the sentiment intensity of a tweet. This score ranges from –1 (most negative) to 1 (most positive), with [–1, –0.1) indicating negative, [–0.1, 0.1] indicating neutral, and (0.1, 1] indicating positive [36].

### Sentiment Trends of the Populations With COPD

**Figure 4:**
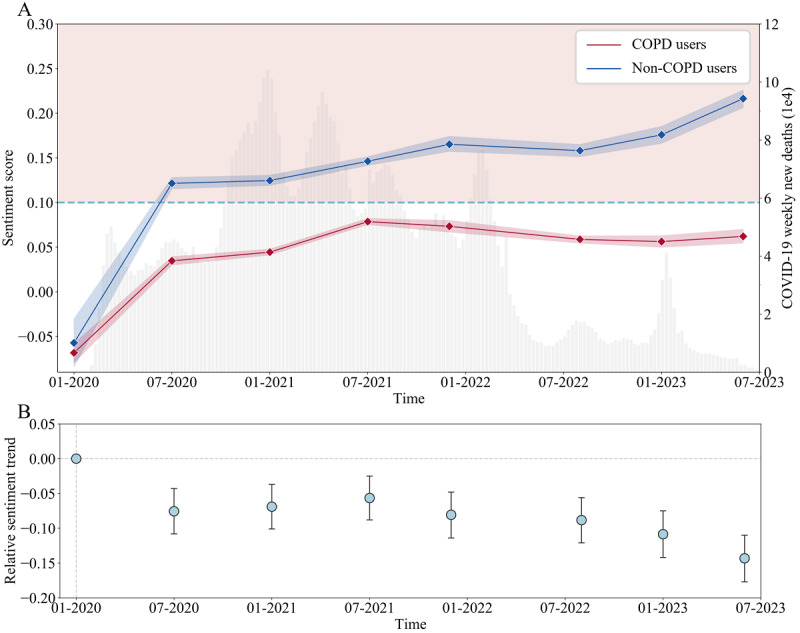
Results of the sentiment trend analysis. (A) Mean sentiment scores with 95% CIs of daily tweets from COPD and non-COPD users over time. The horizontal dashed line (sentiment score = 0.1) is the dividing line between positive (pink area) and neutral (white area). The background bar chart shows the number of COVID-19 weekly deaths. (B) Relative sentiment trends of COPD users, as reflected by the estimated coefficients with 95% CIs calculated by the difference-in-difference model. COPD: chronic obstructive pulmonary disease.

The sentiment scores from both COPD and non-COPD users are shown in Figure 4A, revealing a consistent pattern of lower sentiments in the long term among tweets posted by COPD users compared to those posted by non-COPD users. At the outbreak of COVID-19 in January 2020, the sentiment scores of both the population groups were below –0.05. Between July 2020 and June 2023, the sentiment scores of COPD users remained below 0.1, whereas those of non-COPD users exceeded 0.1. Notably, as the pandemic gradually subsided in 2023, the sentiment scores of non-COPD users increased progressively. In contrast, the sentiment scores of the COPD group still stagnated at a low level.

To accurately understand the relative sentiment trends in the COPD group in contrast with those in the non-COPD group, we employed a DiD model [34] to calculate the estimated coefficients between them. The results calculated by the DiD model can be found in Table S7 in Multimedia Appendix 1. Figure 4B shows that these estimated coefficients remained constantly negative, except for the initial time point, which was set to 0. These coefficients gradually approached 0 until July 2021 but significantly decreased afterward, reaching the lowest point in June 2023. This indicated a continuing relatively downward sentiment trend in the COPD group compared to that in the non-COPD group, and this trend was more pronounced after July 2021.

### Sentiment Disparities Among Different Population Groups

**Figure 5:**
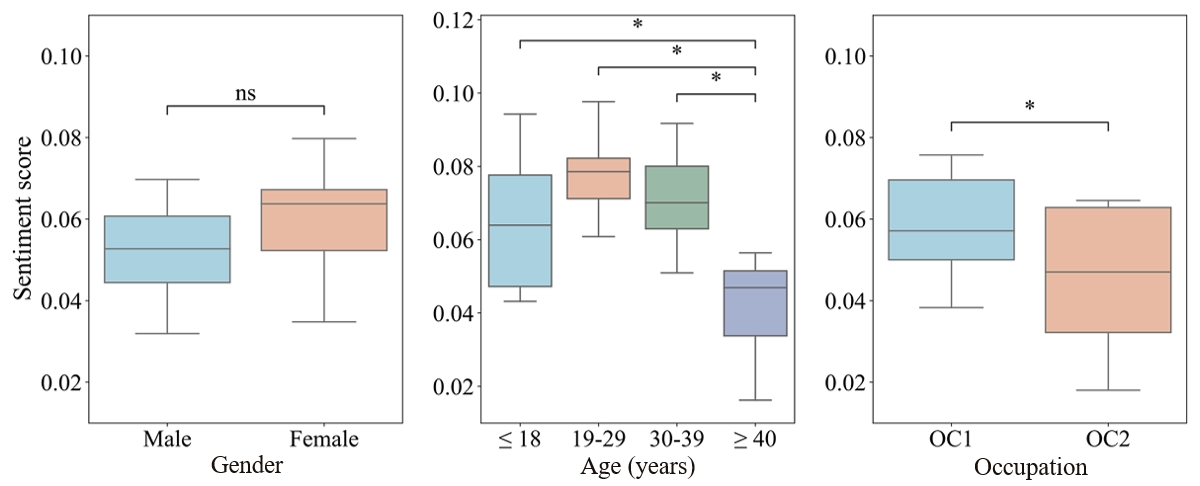
Sentiment comparisons of COPD users among population groups. COPD: chronic obstructive pulmonary disease. OC: occupation category; ns: not significant. *P<.05.

As shown in Figure 5, sentiment scores differ among demographic groups. The average sentiment score of females was higher than that of males; yet, the difference was statistically insignificant (*P*=.08). Among age groups, those aged 40 years and older exhibited much lower sentiments than all the other 3 younger groups. As for the occupation, COPD users with OC2 occupations received lower sentiment scores than those with OC1 occupations (*P*=.03). In short, male COPD users aged 40 years and older with lower socioeconomic status (OC2) had the lowest sentiment in their daily tweets.

To identify emotion patterns in the sentiments of various population groups, we proposed a new method to calculate the values of the sentiment components of Plutchik’s 8 emotions, denoted as sentiment component scores. Detailed descriptions of the emotion pattern method can be found in the Data Analysis Tools. We selected 3 representative timepoints corresponding to the different phases of COVID-19: the initial phase in January 2020, when the pandemic was declared public health emergency of international concern by the World Health Organization; the middle phase in July 2021, when the sentiments of COPD users reached the highest point; and the post-COVID phase in June 2023, after the pandemic was declared over. In the initial phase, fear was the predominant negative emotion among both COPD and non-COPD users. In the middle phase, the sentiment component scores of positive emotions such as joy, trust, and surprise of non-COPD users were significantly higher than those of COPD users. In the post-COVID era, the sentiment component scores of joy and trust of non-COPD users exceeded those of COPD users by far, while the sentiment component score of fear of COPD users became even more negative.

**Figure 6:**
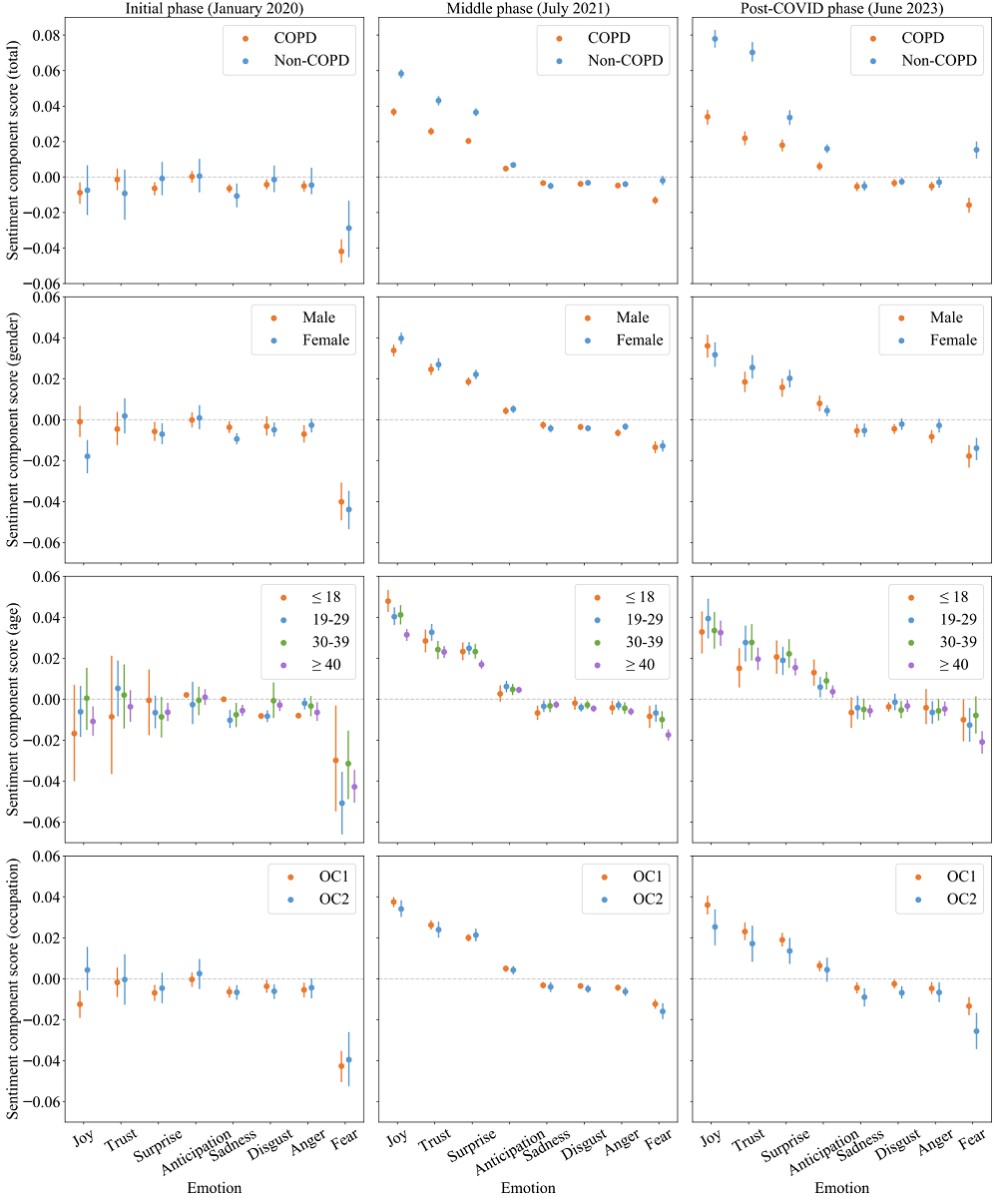
Sentiment component scores of Plutchik’s 8 emotions for different population groups across 3 COVID-19 phases. COPD: chronic obstructive pulmonary disease; OC: occupation category.

As shown in Figure 6, we studied the sentiment component scores of different demographic groups across the 3 COVID-19 phases. The results indicated that females exhibited significantly lower scores of joy than males in the initial phase (January 2020), whereas no obvious difference was observed in the middle phase (July 2021) and post-COVID phase (June 2023). Regarding age, those aged 40 years and older displayed lower sentiment component scores in most emotions, especially fear, compared to those younger than 40 years. As for occupations, those with lower socioeconomic status (OC2) demonstrated lower sentiment component scores than those with higher socioeconomic status (OC1).

## Discussion

### Principal Findings

In this study, we designed a deep learning framework, Deep-COPD, incorporating a series of data retrieval and deep learning methods to investigate the psychological conditions of populations with COPD by using large-scale Twitter data collected during COVID-19 and beyond. First, we computed the attentiveness toward COPD among populations with different demographic features. Subsequently, we identified the popular concerns by hashtags and LDA topics in tweets posted by COPD users in 4 years. Finally, we examined the sentiment trends and prevalent emotions of populations with COPD in contrast to those of populations without COPD.

To the best of our knowledge, this study is the first research effort dedicated to exploring the psychological trends of populations with COPD during and after COVID-19. Unlike in the existing investigations into the psychological impacts of COVID-19 on patients with COPD via conventional survey approaches, where the participant cohorts comprised only a few hundred individuals [10-14], our study intelligently recognized a cohort of 15,347 COPD users as participants. Moreover, by leveraging large-scale Twitter data spanning from January 2020 to June 2023, our study offers invaluable insights into the long-term psychological trends among populations with COPD in the pandemic.

By executing a comparative study between COPD and non-COPD users, this study shows that gender, age, and occupation affected the attentiveness toward COPD. The attentiveness toward COPD was lower in females than in males, significantly higher in adults aged 40 years and older than those younger than 40 years, and significantly higher in individuals of lower socioeconomic status than those of higher socioeconomic status. These interesting findings are aligned with the scientific statistics on the prevalence of COPD. In terms of gender, the prevalence of COPD is reported to be about 5% higher in males than in females [37]. As for age, the prevalence of COPD is appreciably higher in those aged 40 years and older compared to that of COPD in those younger than 40 years, with the incidence progressively increasing with age [38]. For occupation, individuals with lower socioeconomic status (OC2) were more likely to have poorer COPD outcomes than those with higher socioeconomic status (OC1) [8].

Through concern analysis, a notable decline in the popularity of COVID-related concerns among COPD users was observed after 2021. This phenomenon can be attributed to the fact that the SARS-CoV-2 virus undergoes constant mutations. With the emergence of the Omicron variant in November 2021, there has been a decline in the death rate [39]. As more knowledge of the virus has been gained, the associated attention has subsided. Concurrently, a steady rise in health-related concerns among COPD users was noted throughout the course of the pandemic. This trend can be attributed to the higher risk of patients with COPD to more severe diseases under COVID-19 (63%) compared to 33.4% among patients without COPD [8].

By tracking the sentiment trends from January 2020 to June 2023, we found that the sentiments of COPD users were much lower than those of non-COPD users across our study period. Remarkably, through DiD analysis, we noticed an increasingly significant downward trend in sentiments among COPD users compared to non-COPD users after the middle phase of COVID-19 (July 2021). This is an important warning that deserves our close attention. By analyzing the emotion patterns of 3 representative timepoints, this study further demonstrates that negative emotions, including fear, sadness, disgust, and anger, all contributed to the low sentiments of COPD users. Notably, in the post-COVID era (June 2023), COPD users exhibited decreased levels of joy and trust and increased fear compared to their levels of joy and trust during the middle phase of COVID-19. In particular, males, older adults, and individuals with lower socioeconomic status showed heightened fear compared to their counterparts. These results indicated that the populations with COPD experience a much longer impact from COVID-19 than the healthy population.

Last but not the least, similar psychological challenges have also been reported in studies on other chronic conditions under COVID [40-42]. Among all the chronic disease groups studied, including COPD, diabetes, and cardiovascular disease, the patients experienced long-term emotional distress during COVID-19, reinforcing the importance of psychological interventions tailored to different demographic and health characteristics.

### Implications

Considering the implications for practical scenarios, it is clear that patients with COPD need targeted mental intervention and support. The findings of this work reveal a notable surge of fear among COPD users in the post-COVID era, stemming from their health-related concerns. In view of this, we suggest improving the situation in 2 ways to address the emotional challenges faced by patients with COPD. First, offline health care organizations are encouraged to actively offer easy-to-use mental health screenings, interventions, and self-management measures tailored to different population groups in addition to COPD treatments. For example, tools such as the Hospital Anxiety Depression Scale [43] or the Depression Anxiety Stress Scale [44] can be employed to identify patients with COPD who may have mental health concerns, thereby providing suitable guidance and periodic monitoring. Second, by utilizing the potential of social media platforms, web-based health care organizations and influential users can disseminate accessible psychological content, including videos, games, and helpful resources, to raise awareness of mental health and promote positive lifestyles. This approach involves targeted delivery to COPD users according to the distinct demographic and psychological patterns of different groups. By synergizing these offline and web-based approaches, health care organizations and professionals may effectively tailor their intervention measures to alleviate the psychological toll on populations with COPD.

### Limitations

This study has several limitations. First, the identification of COPD users was solely based on self-reported data from Twitter, which may not fully capture all individuals with COPD. Second, this study relies on the VADER tool for sentiment analysis, which, despite its high accuracy, has limitations due to its static lexicon. This restricts its ability to adapt to evolving language trends or capture domain-specific terminology, potentially impacting the accuracy of sentiment classification. Finally, this study is limited to English-language tweets, potentially excluding non–English-speaking populations with COPD and missing cultural variations in psychological responses. These factors should be considered when interpreting the generalizability of our findings. Future research could address these limitations by incorporating data from multiple social media platforms, using more comprehensive methods to verify user health conditions, analyzing multilingual content to capture a broader and more diverse population with COPD, and exploring adaptive or domain-specific sentiment analysis tools to enhance sentiment detection accuracy.

### Conclusions

This study proposes a novel approach to tracking the psychological trends of COPD users via large-scale Twitter mining. This study reveals that although COVID-19 has been declared to be ended, COPD users remain emotionally distressed, with much higher levels of fear than the healthy population. Therefore, strategies for the prevention and treatment of psychological problems in patients with COPD should be tailored to the characteristics of different population groups. The findings in this study offer valuable guidance to health care providers, policymakers, and researchers on the necessary measures to address the psychological needs of patients with COPD in the post-COVID era. The method implemented through our deep learning approach can be easily repurposed to study the psychological effects of other social events from a long-term perspective.

## Data Availability

The source code for the deep learning framework is available at [45]. In compliance with Twitter’s agreements and policy, we can only share data about Twitter user IDs and tweet IDs for academic purposes. These data are available through data access agreements from the corresponding authors.

## Appendix

Multimedia Appendix 1 Supplementary information on the survey of related studies, details of some methods used in this study, and the results of some methods.

Multimedia Appendix 2 STROBE (Strengthening the Reporting of Observational Studies in Epidemiology) guidelines.

## Abbreviations

COPD: chronic obstructive pulmonary disease
DenseNet: dense convolutional network
DiD: difference-in-difference
IMDb: internet movie database
LDA: latent Dirichlet allocation
M3: multimodal, multilingual, and multiattribute
OC: occupation category
OR: odds ratio
STROBE: Strengthening the Reporting of Observational Studies in Epidemiology
VADER: valence aware dictionary and emotional reasoner
