# Supplementary material for: Exploring Psychological Trends in Populations With Chronic Obstructive Pulmonary Disease During COVID-19 and Beyond: Large-Scale Longitudinal Twitter Mining Study
Source: J Med Internet Res. 2025 Mar 5;27:e54543. doi: 10.2196/54543 (PMC11923484; doi:10.2196/54543)
Supplement: Multimedia Appendix 1 [file jmir_v27i1e54543_app1.docx]

Multimedia Appendix 1

1 Related studies

**Table S1.** Available studies on the psychological impacts of the COVID-19 pandemic on patients

with COPD.

| **Reference** | **Target area** | **Study period** | **Method / Means** | **No. of participants** | **Findings about psychological impact** |
| --- | --- | --- | --- | --- | --- |
| Pedrozo-Pupo and Campo-Arias [1] | Santa Marta, Colombia | March 26th, 2020 | Survey / Telephone, electronic questionnaire | 144 | COPD patients presented high COVID-19 perceived stress, post-traumatic stress risk, depression risk, and insomnia risk. |
| Daynes et al [2] | University Hospitals of Leicester, UK | May, 2020 | Survey / Telephone | 131 | A higher burden of symptoms can lead to anxiety and depression but also anxiety and depression may lead to increased severity of symptoms. |
| Hume et al [3] | North East of England | January to March 2020 | Survey / Telephone, questionnaire | 10 | Shielding leaded to heightened feelings of depression and concerns in COPD patients. |
| Sykes et al [4] | Hull University Teaching Hospitals NHS Trust, UK | March 23 and June 1, 2020 | Survey / Verbal interview | 666 | Almost half (48%) COPD patients were reported a negative impact of the pandemic on their mental health. |
| Scarlata et al [5] | Italy | February to May 2020 | Survey / Telephone, video call | 40 | Loneliness more than restrictions themselves affected health status and psycho-cognitive wellbeing in elderly patients with COPD. |

2 Identification of COPD users and tweets

The COPD filter is a case-insensitive pattern implemented as a regular expression consisting of the following COPD keywords taken from medical websites and papers [6,7]. The specific construction process of this regular expression can be found in the build_match_word_regex() function (lines 17-35) of the filter_copd.py file under Stage1 in our GitHub repository [8].

**Table S2.** COPD keywords.

| **COPD keywords** |
| --- |
| chronic obstructive pulmonary disease |
| copd |
| obstructive lung disease |
| chronic bronchitis |
| pulmonary obstructive airflow |
| chronic obstructive airways disease |
| chronic obstructive respiratory disease |

we adopted this COPD filter to identify COPD users and tweets from two places: biography and tweet text, as shown in Table [A2.](#_bookmark9)

**Table S3.** Two examples of finding COPD users and tweets from our Twitter database.

| **Places** | **Example** |
| --- | --- |
| Biography | Expect anti-#Usury, pro-Palestine & misc.\n\nLove Phi, geodesic domes, chemistry, nature. Have #COPD but fighting it with music therapy. Have travelled a bit. |
| Tweet text | I have asthma and COPD. The COPD came from 27 years of cigarette smoking. I still use my inhalers but not near as frequently as I used to when I was a smoker. 👍 Both my lung doctor and my general practitioner approved vaping and we're thrilled that I quit the cigs. |

3 Occupation categories

**Table S4.** New occupation categories in our study and the original occupation categories in the SOC hierarchy.

| **New occupation category (OC)** | **Original occupation category** |
| --- | --- |
| OC1 | C1: managers, directors and senior officials  C2: professional occupations  C3: associate professional and technical occupations |
| OC2 | C4: administrative and secretarial occupations  C5: skilled trades occupations  C6: caring, leisure and other service occupations  C7: sales and customer service occupations  C8: process, plant and machine operatives  C9: elementary occupations |

4 Results of LDA topic modeling

LDA topic modeling was performed on the corpus of tweets collected over the four years. Through grid searching, the optimal numbers of LDA topics are shown in Table S4[.](#_bookmark11)

**Table S5.** Optimal numbers of LDA topics in four years.

| **Year** | **Optimal number of LDA topics** | **Coherence score** |
| --- | --- | --- |
| 2020 | 5 | 0.6897 |
| 2021 | 11 | 0.5047 |
| 2022 | 10 | 0.4587 |
| 2023 | 12 | 0.4436 |

The topics of the corpus are also categorized into six themes: COPD, COVID-19, Health, Politics, Economics, and Others. Table [A5](#_bookmark13) presents the top 5 most prevalent topics over the four years.

**Table S6** Top 5 topics identified by LDA topic modeling in four years

| **Year** | **Rank** | **Topic** | **Theme** | **Ten most common words** |
| --- | --- | --- | --- | --- |
| 2020 | 1 | Lives of patients with COPD | COPD | people, year, lung, asthma, patient, make, day, time, covid, work |
|  | 2 | COVID-19 impact on patients | COVID-19 | covid, heart, test, death, diabetes, disease, die, positive, test positive, dad |
|  | 3 | COVID-19 cases | COVID-19 | week, covid, survive, chemo, uncle, case, drmc, greenville covid, greenville, drmc greenville |
|  | 4 | Delay of mail-order medications | Economics | order, medication, service, thread, change, mail, postal, policy, postal service, delayed |
|  | 5 | COVID-19 preventions for people with breathing problems | COVID-19 | mask, wear, wear mask, breathing, problem, people, lung, breathing problem, woman, joe |
| 2021 | 1 | Effect of smoking to the lung | COPD | smoking, cancer, lung, smoke, smoker, lung cancer, cigarette, friend, quit, die |
|  | 2 | Conditions of patients with lung diseases | COPD | lung, asthma, condition, study, disease, pulmonary, health, chronic, exercise, data |
|  | 3 | COVID-19 deaths | COVID-19 | death, call, covid, video, cancer, prayer, volunteer, trauma, nh |
|  | 4 | Education | Others | child, school, stop, morning, time, give, lie, news, big, life |
|  | 5 | COVID-19 vaccines | COVID-19 | covid, vaccine, heart, disease, risk, die, year, diabetes, cancer, mom |
| 2022 | 1 | Lives of people | Others | life, hope, change, send, late, mom, make, order, folk, drop |
|  | 2 | Healthcare of lung and heart diseases | Health | care, disease, cancer, lung, follow, stroke, include, heart disease, increase, billion |
|  | 3 | Conditions of Patients with COPD | COPD | patient, exacerbation, acute, admit, asthma, respiratory, condition, severe, hospital, sign |
|  | 4 | Work of patients with COPD | COPD | lung, year, day, time, people, work, give, today, asthma, patient |
|  | 5 | Attitudes towards life | Others | good, heart, love, life, death, people, real, state, man, stay |
| 2023 | 1 | COPD treatments | COPD & Health | treatment, year, care, people, respiratory, asthma, day, feel, love, good |
|  | 2 | Healthcare of diseases | Health | great, care, people, good, health, cancer, disease, year, time, heart |
|  | 3 | Health issues with COVID-19 | Health & COVID-19 | health, die, thing, covid, cancer, life, lung, child, care, year |
|  | 4 | Lives of patients with COPD | COPD & Health | life, patient, respiratory, care, people, smoking, asthma, make, covid, child |
|  | 5 | Patients with heart disease | Others | people, make, give, patient, good, heart, severe, cancer, thing, day |

5 Results of the difference-in-difference (DiD) model

**Table S7.** Results of the estimated coefficients calculated by the DiD model.

| **No. (***k***)** | **Time (***t***)** | **Estimated coefficient (***β_k_***)** | **95% confidence interval** | ***p* value** |
| --- | --- | --- | --- | --- |
| 0 | 01-2020 | .. | .. | .. |
| 1 | 07-2020 | -0.0756 | [-0.108, -0.043] | *<*0.001 |
| 2 | 01-2021 | -0.0690 | [-0.101, -0.037] | *<*0.001 |
| 3 | 07-2021 | -0.0565 | [-0.088, -0.025] | *<*0.001 |
| 4 | 12-2021 | -0.0807 | [-0.114, -0.048] | *<*0.001 |
| 5 | 08-2022 | -0.0883 | [-0.121, -0.056] | *<*0.001 |
| 6 | 01-2023 | -0.1085 | [-0.142, -0.075] | *<*0.001 |
| 7 | 06-2023 | -0.1432 | [-0.177, -0.110] | *<*0.001 |

**References**

1. Pedrozo-Pupo JC, Campo-Arias A. Depression, perceived stress related to COVID, post-traumatic stress, and insomnia among asthma and COPD patients during the COVID-19 pandemic. Chronic respiratory disease. 2020;17:1479973120962800.
2. Daynes E, Gerlis C, Briggs-Price S, et al. COPD assessment test for the evaluation of COVID-19 symptoms. Thorax. 2021;76(2):185-7.
3. Hume E, Armstrong M, Manifield J, et al. Impact of COVID-19 shielding on physical activity and quality of life in patients with COPD. Eur Respiratory Soc; 2020.
4. Sykes DL, Faruqi S, Holdsworth L, et al. Impact of COVID-19 on COPD and asthma admissions, and the pandemic from a patient’s perspective. Erj Open Research. 2021;7(1).
5. Scarlata S, Cardaci V, Santangelo C, et al. Distancing measures in COVID-19 pandemic: loneliness, more than physical isolation, affects health status and psycho-cognitive wellbeing in elderly patients with chronic obstructive pulmonary disease. COPD: Journal of Chronic Obstructive Pulmonary Disease. 2021;18(4):443-8.
6. MedlinePlus. Chronic obstructive pulmonary disease (COPD). Available from: <https://medlineplus.gov/ency/article/000091.htm>. [accessed 2024-9-30]
7. Delestre-Levai I, Aliberti S, Almagro M, et al. Patients’ perspectives on bronchiectasis: findings from a social media listening study. ERJ Open Research, 2021, 7(3).
8. COPD_analysis_code. GitHub. 2024. URL: https://github.com/cyzhang87/COPD_analysis_code. [accessed 2024-12-17]
